# Clinicopathological categorization of hydroa vacciniforme-like lymphoproliferative disorder: an analysis of prognostic implications and treatment based on 19 cases

**DOI:** 10.1186/s13000-019-0859-4

**Published:** 2019-07-17

**Authors:** Na Guo, Yueqiong Chen, Yu Wang, Yuhua Huang, Yanfen Feng, Min Li, Huilan Rao

**Affiliations:** 1 State Key Laboratory of Oncology in South China, Collaborative Innovation Center for Cancer Medicine, Guangzhou, Guangdong 510060 People’s Republic of China; 2 Department of Pathology, Sun Yat-sen University Cancer Center, No. 651, Dongfeng Road East, Guangzhou, Guangdong 510060 People’s Republic of China; 3 Hemocyte Morphology Chamber, Sun Yat-sen University Cancer Center, Guangzhou, Guangdong 510060 People’s Republic of China; 4 Department of Medical Oncology, Sun Yat-Sen University Cancer Center, Guangzhou, Guangdong 510060 People’s Republic of China

**Keywords:** Epstein-Barr virus-positive, Hydroa vacciniforme-like lymphoproliferative disorder, Clinicopathologic features, Prognostic factors, Treatment

## Abstract

**Background:**

Hydroa vacciniforme-like lymphoproliferative disorder (HV-LPD) is a cutaneous form of chronic active Epstein-Barr virus (EBV) infection, which occurs mainly in children in Latin America and Asia. It can progress to systemic lymphoma. However, prognostic factors and treatment remain unclear.

**Methods:**

This retrospective study reviewed the clinical, morphologic, immunophenotypical features, and clinical treatment of 19 patients with HV-LPD.

**Results:**

All 19 patients had skin lesions in the face, extremities, or areas unexposed to the sun, including edema, blistering, ulceration, and scarring. The course was slowly progressive and relapsing. Histopathology showed an atypical lymphocytic infiltrate in the dermis and/or subcutaneous tissue. The lesions had a cytotoxic T/NK-cell immunophenotype. Among 19 patients, 7 (37%) exhibited CD4+ T cells, 5 (26%) exhibited CD8+ T cells, and 7 (37%) exhibited CD56+ cells. Of 12 cases with a T-cell phenotype, molecular analyses demonstrated that 7 had monoclonal rearrangements in the T-cell receptor genes. Three cases had an NK-cell phenotype and had polyclonal rearrangements in the TCR genes. All cases were associated with EBV infections. Among 19 patients, 9 (47.4%) received chemotherapy. Only one patient received allogeneic transplantation and EBV-specific cytotoxic T lymphocyte treatment after chemotherapy. That patient was the only one alive without disease at the latest follow up. Nine patients died of systemic lymphoma with disease progression, indicating irreversible process.

**Conclusions:**

This study confirmed that HV-LPD is a broad-spectrum EBV+ lymphoproliferative disorder. It progressed to EBV+ systemic T/NK lymphoma, although some patients had a more indolent, chronic course. Cytopenia, elevated lactate dehydrogenase, destructive-multiorgan involvement, and older age were poor prognostic factors. Only allogeneic transplantation was curative.

## Introduction

Hydroa vacciniforme-like lymphoproliferative disorder (HV-LPD) was nominated and introduced into the 4th revised edition of the WHO classification of Tumors of Hematopoietic and Lymphoid Tissues [1]. HV-LPD is a cutaneous form of Epstein-Barr Virus-positive (EBV+) T/NK lymphoproliferative disease, which occurs in childhood and carries the risk of systemic lymphoma [2, 3]. Other terms have been used previously to describe HV-LPD, including hydroa vacciniforme-like eruption, edematous scarring vasculitic panniculitis, classic and severe hydroa vacciniforme, hydroa-like cutaneous T-cell lymphoma, and hydroa vacciniforme-like lymphoma (HVLL) [3]. The term HVLL was included the 2008 WHO classification of peripheral mature T-cell lymphoma [4]. The nomenclature and definition changed from HVLL to lymphoproliferative disorder, due to its relationship with chronic active EBV (CAEBV) infections and its clinical course.

HV-LPD is extremely rare. It manifests as recurrent skin lesions infiltrated with T cells or NK cells. It is mostly found in children and young adults from Asian and Latin American countries. It is associated with CAEBV infections. It might persist only in genetically predisposed individuals [5–7].

The clinical course of HV-LPD is variable; it lasts up to 10–15 years [8, 9]. In the early stage, skin lesions tend to occur on sun-exposed areas. Lesions comprise recurrent papulovesicles, crusts, and eventually, vacciniform (pox-like) scars, after several weeks. In the late stage, this disorder involves areas unexposed to the sun and becomes more aggressive. Deep ulcerations, necrosis, and facial edema are common. Severe systemic manifestations might be present, such as intermittent fever, lymph node enlargement, and hepatosplenomegaly. When systemic dissemination occurs (hepatosplenomegaly, lymphadenopathy, and bone marrow infiltration), it is best classified as systemic EBV+ T/NK-cell lymphoma [2, 3].

However, it is not always possible to distinguish between HV-LPD and systemic EBV+ T/NK-cell lymphoma, because these disorders often have overlapping clinical and pathological features at the time of presentation and/or during disease progression [10, 11]. Moreover, patients with HV-LPD exhibit variable clinical courses, but often have fatal outcomes. There are no guidelines or consensus opinions to guide clinicians in making a HV-LPD diagnosis, evaluating disease progression, or selecting treatment. Therefore, we retrospectively reviewed 19 cases of HV-LPD, first to characterize further the clinicopathological and molecular features, and second, to propose clinicopathological prognostic factors and potentially effective treatment.

## Materials and methods

### Patient selection

All patients were examined at the Department of Pathology, Sun Yat-sen University Cancer Center, from September 2008 to May 2017. The HV-LPD diagnosis was based on the 4th revised edition of the WHO classification of Tumors of Hematopoietic and Lymphoid Tissues [1]. The HV-LPD diagnosis was also based on standard published clinical and histopathological criteria, immunohistochemistry results, and findings of T-cell receptor (TCR) rearrangements. Tissue specimens were fixed in neutral buffered formalin and embedded in paraffin for processing. Hematoxylin-eosin stained sections, immunohistochemical staining, in situ hybridization for detecting EBV-encoded RNAs (EBERs*)*, and molecular studies were performed at the Department of Pathology.

### Patient follow-up

Of the 19 patients identified, 9 patients had at least one treatment record in the Sun Yat-sen University Cancer Center. Another five patients were treated at other hospitals. Clinical data and follow-up information were obtained from the hospital and from private medical office records. All patients were followed up until May 2017. Disease progression and recurrence were diagnosed based on clinical examinations, imaging assessments, and pathologic examinations. Overall survival (OS) was measured from the initiation of treatment to either the last follow-up or death from disease. Progression-free survival (PFS) was measured from the initiation of treatment to the first indications of disease progression or relapse, death from disease, or the last follow-up.

### Hematoxylin-eosin staining

HV-LPD tissues were formalin-fixed, paraffin-embedded, and stained with hematoxylin and eosin. The histopathological features of the 19 cases were evaluated independently by two pathologists (Y-H H and H-L R).

### Immunohistochemical staining and EBV in situ hybridization

The tissue blocks were cut into 4-μm sections and processed for immunohistochemistry. The lineage of EBV+ cells was determined by staining with antibodies against CD3, CD5, CD4, CD8, CD56, CD30, TIA1, and Ki67. Immunohistochemical stains were performed with an automated immunostainer (Ventana Medical Systems, Tucson, AZ), according to the manufacturer’s protocol. EBV was detected with the EBV Probe In Situ Hybridization Kit (TRIPLEX INTERNATIONAL BIOSCIENCES, CHINA, CO. LTD).

### T-cell clonality studies

Polymerase chain reaction (PCR) was performed to analyze the clonality of T cells. We use the standardized BIOMED-2 clonality assays-ABI Fluorescence Detection (IdentiClone, InVivo Scribe Technologies, San Diego, CA, USA) according to the manufacturer’s instructions. PCR products were analysed by capillary electrophoresis using an ABI 3500XL genetic analyser (Applied Biosystems,Foster City, CA, USA). DNA quality was assessed through amplification of a control gene size ladder.

First, after deparaffinization and proteinase K digestion, DNA was extracted from 10-mm paraffin sections, according to the standard procedure. Then, T-cell clonal expansion was detected by analyzing rearrangements in the TCR beta chain (Vb-Jb and Db-J-b) and TCR genes. Positive and negative controls were set appropriately in all experiments. Clonality was assessed according to well-established recommendations.

### EBV DNA blood load measurement

Total plasma cell-free DNA was isolated with the QIAamp Blood Mini Kit (QIAgen, Inc., Valencia, CA, USA), according to the manufacturer’s protocol. Plasma levels of EBV DNA toward the BamHI-W region were determined with the quantitative PCR method. The primers were designed as previously reported [12]. DNA from EBV-negative healthy volunteers comprised one negative control, and another negative control was run with no template. Controls were included on each plate. Results are expressed as the number of EBV copies/ml plasma. Zero copies/ml was selected for the cut-off value in evaluating EBV DNA levels.

There was a positive control used for EBV DNA detection each time. To estimate the sensitivity of the PCR assay, gradient dilutions of the synthetic EBV fragment plasmid were prepared and amplified by the PCR assay. Using absolute quantification, a standard curve was made by 10^3^, 10^4^, 10^5^ and 10^6^. And 10^3^ and 10^5^ were used as positive controls .

### Statistical analysis

Data are described as the mean ± standard deviation (SD), range, frequency (number of cases), and percentages, when appropriate. All statistical calculations were performed with the Statistical Package for Social Sciences software, version 20 (SPSS, Inc. Chicago, IL, USA).

## Results

### Clinical characteristics

Nineteen cases were diagnosed in our center from September 2008 to May 2017. The incidence of HV-LPD was 1.9% among patients with mature peripheral T-cell lymphoma in our center [13]. The cohort comprised 9 males and 10 females, aged 2 to 21 years (median 8 years). All patients were ethnically Chinese. The clinicopathological findings of our cases are summarized in Table 1.

**Table 1:** Clinicopathologic features of patients with HV-LPD

Case	Age(y)/ Sex/History (y)	Skin lesions/location	Systemic manifestations	Size of atypical cells/infiltration depth/necrosis	LN, BM biopsy	LDH (IU/L)	Phenotype	Therapy	Best response to treatment	Current status	Survival after Diagnosis
1	9/F/1	PUCS/face, scalp, extremities	FHSL	Medium/dermis	EBV+ cells in interfollicular area of LN	324	CD8	Chemotherapy (IMR) and Radiotherapy. Maintained herbal remedy	CR	AWD	8.7Y
2	13/F/3	Erythema, PUCS/face, extremities, and areas not exposed to sun	FHSL	Medium/dermis and fatty tissue/Fatty tissue necrosis	EBV+ cells diffusely infiltrated LN	624	CD56	Chemotherapy (NHL-BFM-90) for 3 cycles [14]	PD	DOD	1.1Y
3	8/M/1	PUCS, nodules/face, extremities	FHSL	Medium/dermis	EBV+ cells present in interfollicular area of LN	450	CD56	Chemotherapy (MTX + VP-16) and Radiotherapy	PR	AWD	7.6Y
4	4/M/1	PUCS/ areas exposed to sun; HMB	Fever	Small and medium/dermis and subcutaneous tissue	ND	NA	CD4	Herbal remedy	PD	AWD	6Y
5	5/M/1	PUCS, nodules/face, extremities	FHS	Small and medium/dermis	ND	NA	CD8	Chemotherapy (IMR) and Radiotherapy. Maintained Herbal remedy	PD	DOD	4.4Y
6	8/F/1.5	PUCS/face, arms, areas not exposed to sun	FHSL	Medium and large/dermis	ND	NA	CD56	NA	NA	DOD	3Y
7	10/M/3	PUCS/face, extremities, HMB	FL	Small and medium/dermis and subcutaneous tissue	ND	298.4	CD8	Chemotherapy (NHL-BFM-95) for 6 cycles [15]	PR	DOD	2.2Y
8	2/F/0.5	PUCS, nodules/face, extremities	None	Medium/dermis and subcutaneous tissue	ND	NA	CD56	NA	NA	LFU	LFU
9	21/M/14	PUCS/areas exposed and unexposed to sun; HMB	FSHLNP nodules, HPS	Medium and large/dermis and subcutaneous tissue	EBV+ cells diffusely infiltrated LN and BM	1202	CD4	Chemotherapy (GEM+VP16 + MTX + P-ASP+thalidomide for 6 cycles)	PR	DOD	0.6Y
10	7/F/1	PUCS/face, extremities,	Fever	Medium/dermis and fatty tissue	ND	NA	CD8	NA	NA	LFU	LFU
11	5/M/2	PUCS, nodules/face, extremities, trunk; HMB	FHSL	Medium/dermis	EBV+ cells in interfollicular area of LN	318	CD4	Chemotherapy (NHL-BFM-95) for 6 cycles; Sibling HSCT; and EBV-CTL treatment [15]	CR	Alive without disease	4.4Y
12	13/F/1	PUCS/face, extremities, trunk, and oral cavity	FL	Medium/dermis	BM-	482	CD56	Chemotherapy (P-ASP+GEM+L-OHP) Maintained herbal remedy	PD	DOD	2.2Y
13	12/F/2	PUCS, nodules/face, trunk, and extremities	FHSL	Medium/dermis and fatty tissue	EBV+ cells diffusely infiltrated LN and BM	358.9	CD56	Chemotherapy (GEM for 6 cycles) and antivirus treatment (Ganciclovir)	CR	AWD	3.2Y
14	5/M/3	PUCS/face, extremities, trunk	FL	Medium/dermis and subcutaneous tissue	BM-	334.6	CD4	Chemotherapy (First Line: GEM for 6 cycles, Second Line: VP-16 + VCR for 1 cycle, Third Line: VLB + PDN for 1 cycle)	PR	AWD	2.9Y
15	7/F/4	PUCS/face, extremities	FL	Medium/dermis	BM-	323.2	CD56	Chemotherapy (GEM for 6 cycles)	CR	DOD	1.3Y
16	12/F/4	PUCS/face, trunk, arms and legs; HMB	FHSL	Medium/dermis and fatty tissue	BM-	NA	CD4	NA	NA	DOD	0.7Y
17	11/M/9	PUCS, nodules/face, arms, legs, and areas not exposed to sun; HMB	FHSL	Medium/dermis and subcutaneous tissue	EBV+ cells diffusely infiltrated LN and BM	585	CD4	Chemotherapy (First Line: NHL-BFM-95, Second Line: GEM for 2 cycles) [15]	PD	DOD	2.4Y
18	8/M/2	PUCS/face, arms, legs, and trunk	Fever	Medium/dermis and subcutaneous tissue	BM-	312	CD4	Chemotherapy (methylprednisolone, azithromycin, acitretin, and cyclosporine)	PR	AWD	2.2Y
19	F/5/3	PUCS/face, legs, and trunk	Fever	Medium/dermis and subcutaneous tissue	ND	NA	CD8	NA	NA	LFU	LFU

*PUCS* papulovesicles, ulcers, crust, scars, *HMB* Hypersensitivity to mosquito bites, *FHSL* fever, hepatosplenomegaly, lymphadnopathy, *FHS* fever, hepatosplenomegaly, *FL* fever, lymphadnopathy, *NP nodules* nasopharynx nodules, *HPS* hemophagocytic syndrome, *LN* lymph node, *BM* bone marrow, *BM*- BM not involved, *AWD* Alive with disease, *DOD* dead of disease, *NA* not applicable, *ND* not done, *LFU* lost to follow-up, *IMR* interferon, mechlorethamine, and retinoic acid, *NHL* Non Hodgkin lymphoma, *BFM* Berlin-Frankfurt-Münster, *MTX* methotrexate, *VP-16* etoposide, *P-ASP* pegaspargase, *HSCT* hematopoietic stem cell transplantation, *GEM* gemcitabine, *L-OHP* oxaliplatin, *VCR* vincristine, *VLB* vinblastine, *PDN* prednisone, *CR* complete remission, *PD* progressive disease, *PR* partial remission

**Fig. 1:**
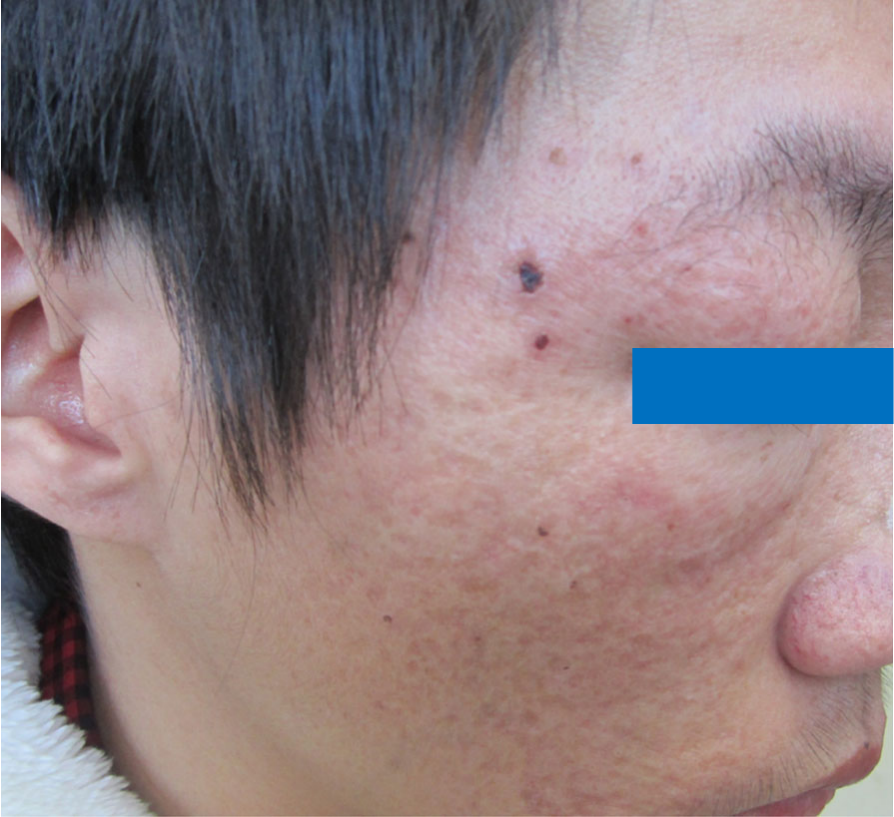
Case 9 shows an ulcerated lesion and vacciniform scar on the face. Note the periorbital edema

The main clinical features were papules (*n* = 19/19, 100%,) and fever (*n* = 18/19, 94.7%). Only one patient (case 8) had no B symptom at the diagnosis. All lesions were located in the skin, and they included papulovesicles, blisters, ulceration, and scars. Eruptions were seasonal; they typically occurred in spring or summer. Eighteen patients had recurrent and slowly progressive, relapsing clinical courses. One patient (case 19) presented with a severe HV. In the early stage, most skin lesions initially occurred after sun exposure and subsided after sun protection. The lesions typically appeared on the face and extremities. The skin lesions typically healed within 1–2 weeks, with vacciniform scarring, and they recurred in the same distribution. Rarely, cases presented with fever at the beginning. As the disease progressed, 15 patients (88%) presented with severe systemic manifestations, including intermittent fever, wasting, lymphadenopathy, and hepatosplenomegaly. Severe skin lesions presented as deep ulcers, necrosis, plaques, or face edema in these patients (Fig. 1). The ulcerations took a longer time to heal, and they left scars.

Six cases had a history of mosquito-bite hypersensitivity. Among these, four had a CD4+ phenotype, and the other two had a CD8+ phenotype. No patient was hypersensitive to light.

The available routine laboratory tests revealed that all patients (11/11) had elevated lactate dehydrogenase (LDH) levels (range 312 to 1202 IU/L), when they were initially diagnosed with HV-LPD. Available data for seven patients showed positive EBV DNA blood loads in all patients (*n* = 7/7). In the 9 patients who had the treatment record in Sun Yat-sen University Cancer Center, the range of time-to progression were from 0.2 to 7 years (median, 0.63 yrs). Both the LDH and EBV DNA values at disease progression or the last follow-up were shown in Table 2. Both of the LDH and EBV DNA values, the important index for lymphoma, reflected the effect of the treatment. During the treatments, both of the LDH and EBV DNA loads declined. However, both of the LDH and DNA loads rose to high levels when the disease progressed (Table 2).

**Table 2:** EBV DNA loads and LDH level in the 9 patients who had the treatment record in Sun Yat-sen University Cancer Center

Case	EBV DNA (copy/ml)			LDH (IU/L)			Time-to-progression	Current status
	Before treatment	The minimum level during treatment	Progression/last follow-up	Before treatment	The minimum level during treatment	Progression/ last follow-up		
Case1	ND	ND	ND	324	ND	207.7	7.0y	AWD
Case2	ND	ND	ND	624	215	315.7	0.2y	DOD
Case 7	1.88 × 10^8^	6.63 × 10^5^	1.33 × 10^6^	298.4	206.2	888	0.8y	DOD
Case 9	5.62 × 10^6^	2.0 × 10^1^	6.83 × 10^4^	1202	248.7	1166	0.5y	DOD
Case 11	5.68 × 10^6^	6.93 × 10^4^	5.95 × 10^4^	318	179.6	259.8	NA	Alive without disease
Case 13	1.70 × 10^5^	6.0 × 10^4^	2.69 × 10^3^	358.9	238.6	248.9	2.1y	AWD
Case 14	3.95 × 10^5^	6.59 × 10^3^	4.53 × 10^4^	334.6	203.7	276.3	0.3y	AWD
Case 15	6.94 × 10^5^	1.20 × 10^3^	7.56 × 10^4^	323.2	251.7	624.4	0.75y	DOD
Case 17	1.58 × 10^5^	2.25 × 10^4^	3.09 × 10^5^	585	119.4	363.9	0.25y	DOD

*AWD* Alive with disease, *DOD* dead of disease, *NA* not applicable, *ND* not done

### Histopathology

**Fig. 2:**
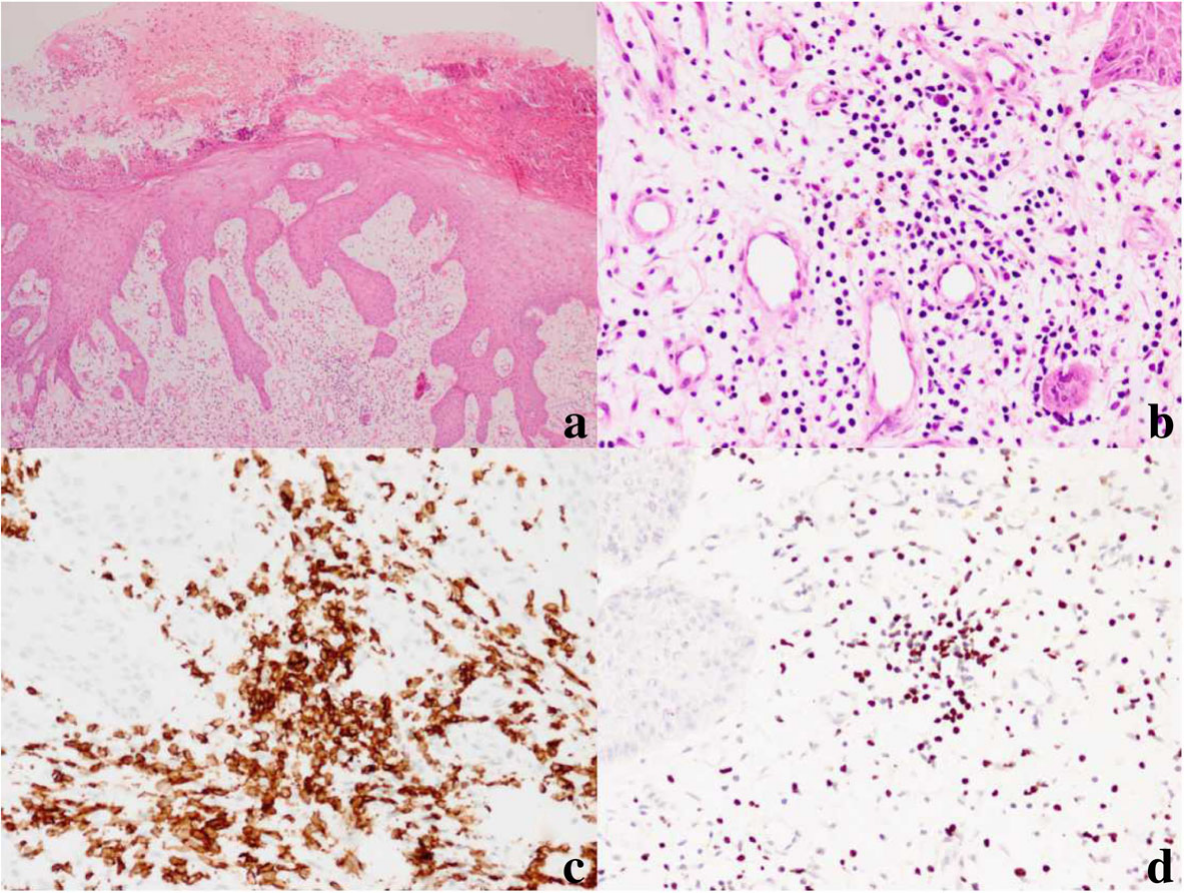
HV-LPD morphology and immunophenotype of case 10. **a** Skin biopsy shows edema and vesicles in the epidermis (hematoxylin and eosin [H&E], 40×). **b** Skin biopsy shows an infiltration of atypical lymphocytes surrounding adnexa and blood vessels (H&E, 200×). **c** Immunohistochemical CD8 stain shows that the cells surrounding the adnexa and blood vessels are strongly CD8-positive (200×). **d** In situ hybridization for EBV-encoded RNA showed positive signals in infiltrating lymphocytes (200×)

**Fig. 3:**
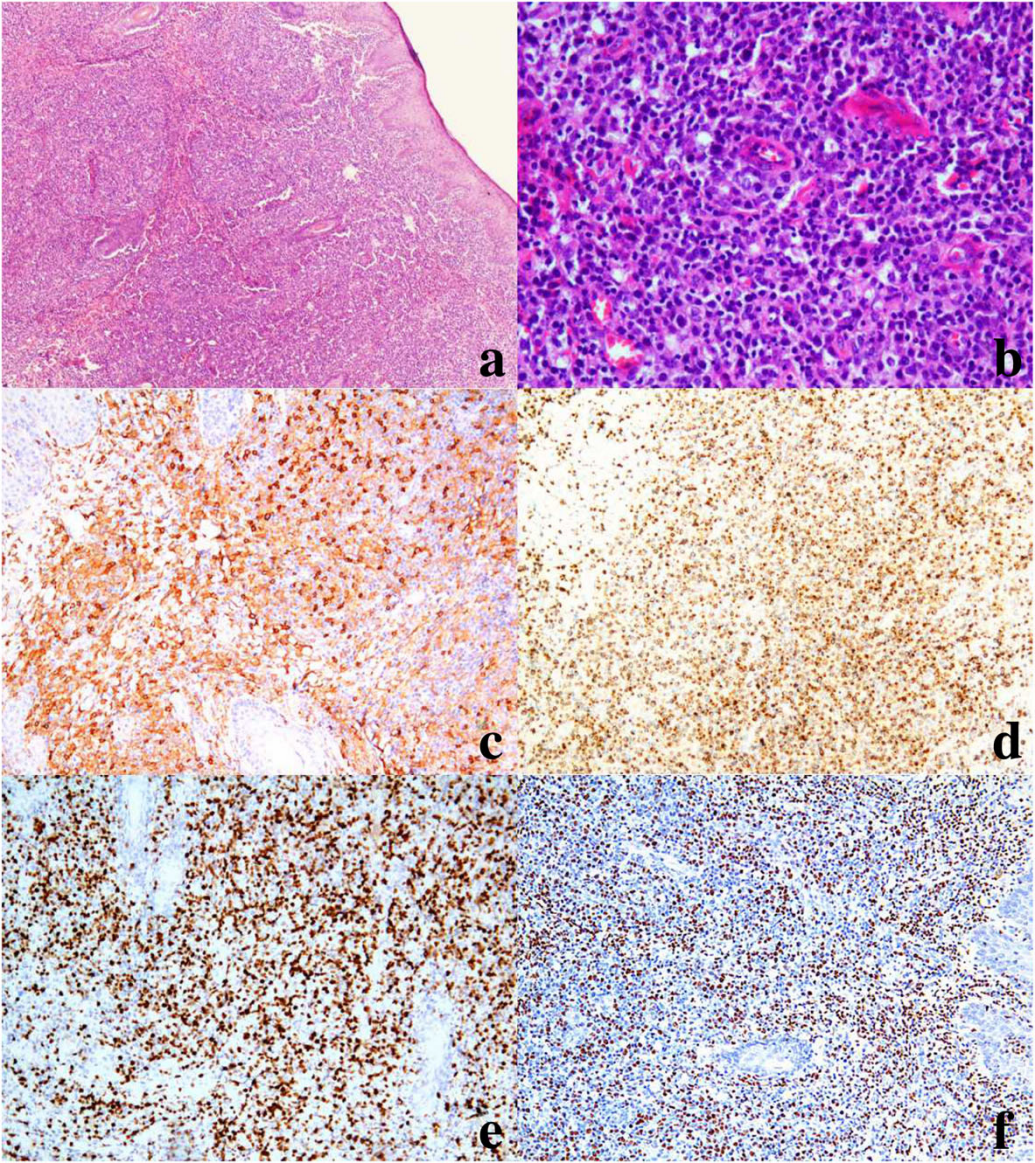
Morphology and immunophenotype of case 9, which progressed to systemic T-cell lymphoma. **a** Skin biopsy shows a diffuse, atypical lymphocyte infiltrate, with a marked epidermis and subcutaneous distribution (hematoxylin and eosin stain [H&E], 40×). **b** Skin biopsy shows medium- to large-sized lymphocytes, with enlarged, oval and pleomorphic nuclei (H&E, 200×). **c** Strong staining of CD4+ cells (IHC, 200×). **d** GranB Positive cells (IHC 100×). **e** Ki67 expression is high in 70% of tumor cells (IHC 100×). **f** In situ hybridization shows that neoplastic cells are positive for EBV-encoded RNA (100×)

Histologically, all the biopsies featured infiltration of atypical lymphoid cells surrounding blood vessels and skin appendages in the dermis. In 12 cases (63%), atypical lymphocytes had infiltrated deep into fatty tissue or subcutaneous tissue. They were small to intermediate in size, and they had enlarged, irregular nuclei with nucleoli, admixed with an inflammatory background composed of small lymphocytes, plasma cells, histocytes, and eosinophils (Fig. 2). Interestingly, nine cases developed into systemic lymphoma; these displayed dense and diffuse, atypical, medium- or large-sized lymphocytes (Fig. 3). Mitoses were brisk. Tumor necrosis was observed. However, we observed no angio-invasion or vascular destruction in any of the nine cases analyzed.

Seven patients had lymph node biopsies. Four biopsies showed preserved architecture with open sinuses and paracortical hyperplasia. Cells that had infiltrated the lymph node were small- and medium-sized, with irregular nuclei and inconspicuous nucleoli. Cases 2, 9, and 17 progressed to systemic NK/T cell lymphoma. They exhibited diffuse effacement of the normal nodal architecture, due to the infiltration of homogeneous medium and large mixed lymphocytes with hyperchromatic nuclei and irregular nuclear contours. Cases 9, 13, and 17 harbored bone marrow that had been infiltrated by atypical lymphocytes.

### Immunohistochemical studies and EBERs

The immunohistochemical findings are summarized in Table 3. The lymphoid cells that had infiltrated under the epidermis were 100% positive for CD3 and TIA1, and partially expressed CD30. With in situ hybridization, all cases displayed the presence of EBERs. Cells that carried EBERs were concentrated mainly around blood vessels and adnexa in the dermis (Figs. 2 and 3), but also in subcutaneous tissue.

**Table 3:** Immunophenotype and molecular analysis of biopsies from 19 patients with HV-LPD

Case	CD3	CD8	CD4	CD56	TIA1	CD30	Ki67	EBERs	TCR rearrangement
1	**+**	**+**	**–**	**–**	**+**	**–**	30%	**+**	**–**
2	**+**	**–**	**–**	**+**	**+**	**–**	60%	**+**	**–**
3	**+**	**–**	**–**	**+**	**+**	**–**	30%	**+**	ND
4	**+**	**–**	**+**	**–**	**+**	P+	80%	**+**	**–**
5	**+**	**+**	**–**	**–**	**+**	ND	30%	**+**	**–**
6	**+**	**–**	**–**	**+**	**+**	ND	30%	**+**	–
7	**+**	**+**	**–**	**–**	**+**	**–**	30%	**+**	**–**
8	**+**	**–**	**–**	**+**	**+**	P+	50%	**+**	ND
9	**+**	**–**	**+**	**–**	**+**	**+**	70%	**+**	+
10	**+**	**+**	**–**	**–**	**+**	ND	20%	**+**	+
11	**+**	**+**	**–**	**–**	**+**	**–**	30%	**+**	+
12	**+**	**–**	**–**	**+**	**+**	**–**	30%	**+**	ND
13	**+**	**–**	**–**	**+**	**+**	**+**	70%	**+**	F
14	**+**	**–**	**+**	**–**	**+**	ND	40%	**+**	+
15	**+**	**–**	**–**	**+**	**+**	**–**	60%	**+**	**–**
16	**+**	**–**	**+**	**–**	**+**	P+	70%	**+**	**+**
17	**+**	**–**	**+**	**–**	**+**	**+**	40%	**+**	**+**
18	**+**	**–**	**+**	**–**	**+**	**–**	10%	**+**	**+**
19	**+**	**–**	**+**	**–**	**+**	**–**	60%	**+**	**–**

*EBERs* EBV-encoded RNAs, *TCR* T-cell receptor, *P* partial, *ND* not done, *F* failure to extract sample DNA

Of 19 patients, five biopsies (26%) had CD8+ T cells, but no CD4+ or CD56+ cells (Fig. 2c). Seven (37%) had initial biopsies with CD4+ T cells, but no CD8+ T cells or CD56+ cells (Fig. 3c). Seven biopsies (37%) had CD56+ cells, but no CD4+ or CD8+ T cells, which indicated an NK-cell phenotype.

### Molecular studies

Molecular analyses demonstrated that 7 of the 12 biopsies with a T-cell phenotype had undergone monoclonal rearrangements in the TCR genes. These seven cases were originally classified as HVLL, based on the monoclonality of the TCR gene rearrangements and fulfillment of the 2008 WHO classification criteria. The three biopsies with an NK-cell phenotype lacked detectable clonal rearrangement.

### Treatment and outcome

Due to the long clinical course, the patients received a variety of treatments. Most patients were primarily treated with immunomodulating or immunosuppressive therapies, such as steroid cream or interferon, mechlorethamine, retinoic acid, etc. In the early stage, skin lesions typically improved with these treatments, but later, these treatments lost some efficacy.

Among the 19 patients, nine (47.4%) received chemotherapy, one patient (5.3%) received an herbal remedy, one patient (5.3%) received a combination of chemotherapy and radiotherapy, one patient (5.3%) received a combination of chemotherapy and herbal remedy, and two patients (10.5%) received sequential chemotherapy, radiotherapy, and herbal remedy. The other five patients (26.3%) received no anti-tumor treatment. The main chemotherapy regimens in our study were BFM-90/95, for children with non-Hodgkin lymphoma (NHL), and gemcitabine for adults with extranodal NK/T-cell lymphoma, nasal type. Only one patient (case 11) in our study received an allogeneic hematopoietic stem cell transplantation and EBV-specific cytotoxic T lymphocyte (EBV-CTL) treatment after chemotherapy.

Among the 14 patients that received treatment, four patients (28.6%) achieved complete remission (CR), five patients (35.7%) achieved partial remission (PR), and five patients (35.7%) had progressive disease. Among the latter, four patients developed systemic lymphoma and died of disease during the anti-tumor treatment. Three patients died of disease during the follow-up with the best effect of CR or PR. The 2-year, 3-year and 5-year OS was78.6, 56.3 and 45.0%, respectively.

Among all of the19 patients in our study, the 2-year, 3-year and 5-year OS were 75.0, 47.6 and 38.1%, respectively, excluding 3 patients who lost to the follow up. Nine patients developed systemic lymphoma and died of disease with or without treatment.

### Differences between patients with T-cell and NK-cell phenotypes

**Fig. 4:**
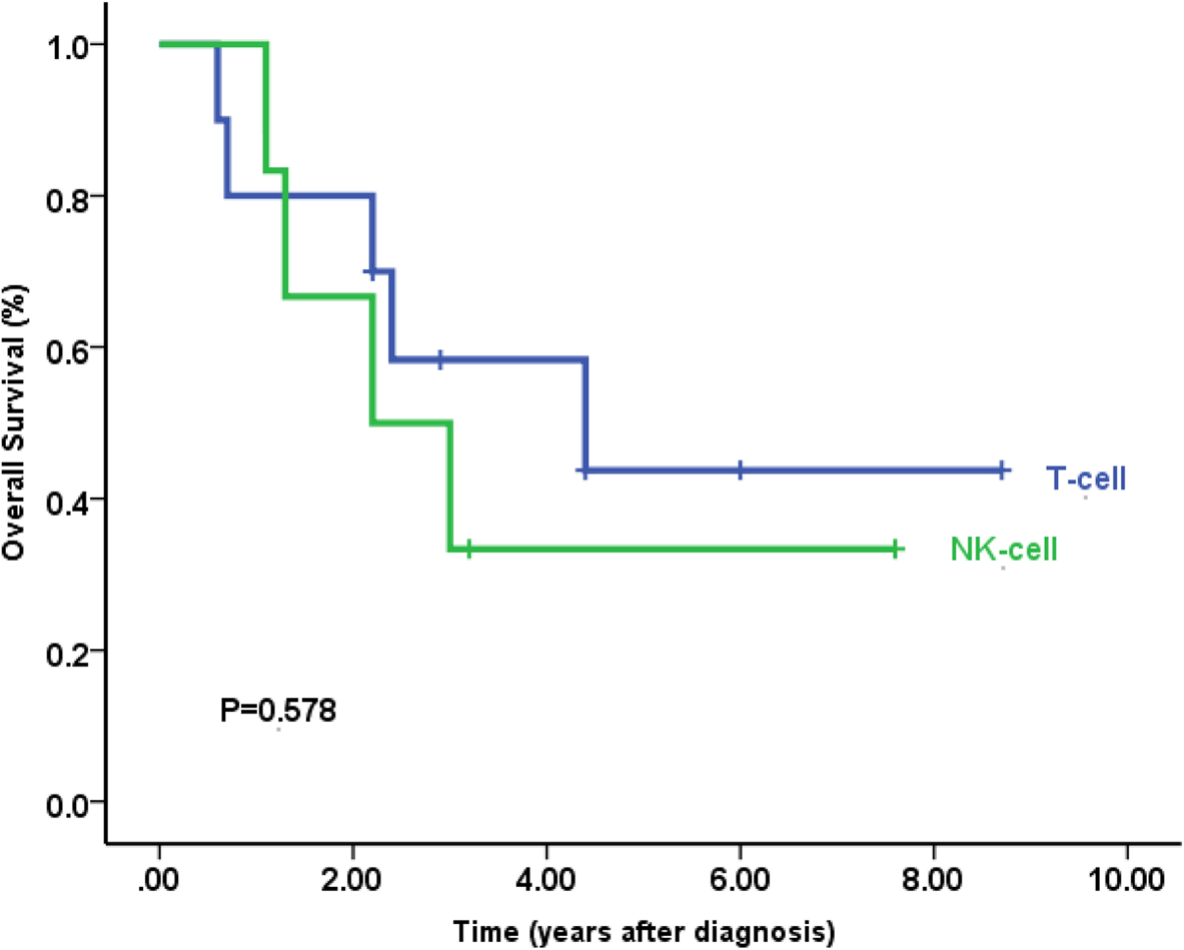
Kaplan-Meier survival curves for patients with HV-LPD, categorized by a clinicopathological feature. Patients with T-cell lineage disease (CD4 and CD8) had similar clinical outcomes with those of NK-cell lineage disease for all the patients with or without the treatment (*n* = 19, *p* = 0.578 for T-cell vs. NK-cell). Survival-time differences were analyzed with the log-rank test

We compared the clinical features between patients with T-cell and NK-cell phenotypes. The male-to-female ratios were quite different (T cell: 2:1 vs. NK cell: 1:6), showing female dominance in NK phenotype. But the median ages were similar between groups (7.5 vs. 8 years). The two groups had similar skin lesions, and both had systemic symptoms at presentation. HMB was documented only for the T-cell phenotype, including five patients with a CD4+ T-cell phenotype and one patient with a CD8+ T-cell phenotype. Progression to T/NK-cell lymphoma in the nasal region occurred in one patient (case 9) with a CD4+ T-cell phenotype. The two groups had similar incidences of systemic lymphoma, and the clinical outcomes were similar in all patients with T-cell lineage disease (CD4+ and CD8+) and those with NK-cell lineage disease (*p* = 0.578 for T-cell vs. NK-cell, Fig. 4).

## Discussion

HV-LPD is extremely rare in Western countries. Most studies described recurrent skin lesions with T-cell or NK-cell phenotypes in children and adolescents from Asian and Latin American countries [11, 16, 17]. South China has a high-incidence of EBV-associated nasopharyngeal carcinoma and extranodal NK/T-cell lymphoma, nasal type [13]. In our previous study, HV-LPD comprised 8.1% of peripheral T/NK-cell neoplasms in children and adolescents [18], indicating the prevalence of specific EBV strains and genetic background or genetic predisposition, a hypothesis that requires further study for confirmation.

In our study, all cases of HV-LPD had undergone an initial smoldering stage that lasted from 1 to 10 years. The early stage clinical features fit the criteria for hydroa vacciniforme. All patients had self-limited vesiculopapular eruptions on exposed areas, including the face, dorsal surfaces of the hands, and lower limbs, with no manifestation or only mild fever. As the disease progressed, most patients exhibited severe, extensive skin lesions with systemic manifestations, including fever, hepatosplenomegaly, and lymphadenopathy, consistent with other reports [2, 19, 20]. The term HV-LPD changed from hydroa vacciniforme-like lymphoma to lymphoproliferative disorder by the 4th revised edition of WHO classifications would be a more appropriate classification and a more descriptive term [1, 21, 22].

The histopathology of biopsies from 11 patients showed that atypical lymphocytes had infiltrated, and subcutaneous tissues were involved. Nine patients died of systemic T/NK lymphoma. All had elevated LDH levels (range: 318 to 1202 IU/L) and higher EBV DNA blood loads. Our study confirmed previous results that indicated that HV-LPD and HVLL groups comprised a spectrum of cutaneous CAEBV forms. HV-LPD is an EBV-associated lymphoproliferative disorder of T or NK-cell phenotype with risk for progression to HVLL.

There is no method in the literature for distinguishing between HV-LPD and progression to systemic NK/T cell lymphoma. This lack of distinction often confuses pathologists and chemotherapy physicians. The challenge remains to identify morphological or clinical markers for predicting which patients are at high risk of progressing to systemic lymphoma. We found that the presence of a TCR gene rearrangement was not useful for making a diagnosis of lymphoma, because, in our study, case 14 and 18 had monoclonalities in the early stage. Four patients developed systemic lymphoma and died of the disease progression during chemotherapy treatment. As shown in Table 1, all those patients displayed atypical, medium- to large-sized cells with deep skin ulcers, highly elevated LDH, long-lasting high EBV DNA loads (typically > 1 × 10^5^ copies), severe systemic manifestations, diffuse effacement of normal nodal architecture by atypical cell infiltration, and bone marrow involvement. Older patients tended to develop lymphoma, which was probably associated with an irreversible process [23].

HV-LPD was originally thought to be derived from skin-homing cytotoxic T-cells or NK cells. The majority of studies on HV-LPD reported a T-cell phenotype with divergent immunoexpression of either CD4 or CD8 [24, 25]. However, EBV-infected cells might be CD4+ T cells, CD8+ T cells, or NK cells. EBV-infected cellular lineages have varied in different studies and populations. Doeden et al. described patients with a NK-cell phenotype [6]. Interestingly, our cases exhibited the CD4+ phenotype (7/19), the CD8+ phenotype (5/19), and the NK-cell phenotype (7/19). The clinical course of the CD56 phenotype was not more aggressive than that of the T-cell phenotype; only two cases died of systemic NK lymphoma. The other two patients with the CD56 phenotype had longer clinical courses, and were alive with disease after 3 or 7 years of follow up. This finding was consistent with findings in previous reports [5]. Doeden et al. described two boys with the CD56 phenotype that exhibited an indolent disease course. In the present study, T-cell or NK-cell lineages were not identified as independent adverse prognostic factors. However, the sample size was quite small; in future, a larger sample is needed to analyze the association between phenotype and disease course or outcome.

Currently, there is no standard therapy for patients with HV-LPD. Several treatments for HV-LPD have been suggested, including antiviral agents, anti-inflammatory agents, radiotherapy, chemotherapy, EBV-specific cytotoxic T-cell regimen, bone marrow or allogeneic hematopoietic stem cell transplantation, and immunomodulative therapies, such as interferon-a or interleukin-2 [5, 26–28]. Chemotherapy and/or immunomodulative therapies have been used to treat many patients in progressing stages, but they provided only temporary remission or limited benefit.

In this study, the main regimens of chemotherapy were BFM-90/95, for pediatric non-Hodgkin lymphoma (NHL), and gemcitabine, for adult extranodal NK/T-cell lymphoma, nasal type. The effects were typically transient, and they did not result in persistent remission. These treatments killed the EBV-infected cells, but they could not prevent patients from EBV re-infection. Only one patient (No. 11) received allogeneic hematopoietic stem cell transplantation and EBV-CTL treatment after chemotherapy, and that patient was the only one alive without disease at the latest follow up. Thus, allogeneic HSCT appeared to be the only curative treatment for HV-LPD. This result was consistent with those from previous reports [29, 30].

In summary, we demonstrated that HVL-LPD had specific features that could be classified into different clinicopathological categories; this finding might potentially impact future clinical treatments and outcomes. We established the diagnosis of systemic EBV+ T/NK cell lymphoma as a result of HV-LPD progression, based on the clinical, histopathological, and immunohistochemical features, in addition to EBV detection. We showed that EBV-DNA loads, LDH, cytopenia, and multiorgan destructive involvement were poor independent prognostic factors, distinct from findings in previous studies. The monoclonality of TCR gene rearrangements could be a referential index for evaluating HV-LPD progression. Future studies on genetic abnormalities might contribute to our understanding of the biological behavior of HVL-LPD and to establishing a therapeutic strategy for patients.

## Data Availability

The datasets generated and/or analyzed in this study are available from the corresponding author upon reasonable request.

## Abbreviations

CAEBV: Chronic active EBV
EBV: Epstein-Barr virus
EBV-CTL: EBV-specific cytotoxic T lymphocyte
HVLL: Hydroa vacciniforme-like lymphoma
HV-LPD: Hydroa vacciniforme-like lymphoproliferative disorder
NHL-BFM-90/95: The Berlin-Frankfurt-Munster Group Trial NHL-BFM 90/95 regimen for the treatment of the children and adolescents with Non-Hodgkin’s lymphomas
OS: Overall survival
PFS: Progression-free survival
